# Enhancement of spinal cord injury repair in rats using photo‐crosslinked GelMA hydrogel combined with mitochondrial transplantation

**DOI:** 10.1002/btm2.70040

**Published:** 2025-07-03

**Authors:** Wenyong Gao, Can Tang, Diancheng Hang, Hao Chen, Dongsheng Li, Youjie Yan, Wuchang Wang, Hao Peng, Xianmin Wang, Enpeng Zhang, Min Wei, Hengzhu Zhang, Xiaodong Wang

**Affiliations:** ^1^ Department of Neurosurgery Northern Jiangsu People's Hospital Yangzhou China; ^2^ Department of Neurosurgery The Yangzhou School of Clinical Medicine of Dalian Medical University Yangzhou China; ^3^ People's Hospital of Jiawang District Yangzhou China; ^4^ Department of Emergency Medicine Xi'an Honghui Hospital Xian China; ^5^ Department of Neurological Function Testing Northern Jiangsu People's Hospital Yangzhou China

**Keywords:** GelMA hydrogel, mitochondrial transplantation, spinal cord injury

## Abstract

Acute spinal cord injury (SCI) induces mitochondrial oxidative stress, cellular bioenergetic crises, impaired protein degradation, and subsequent degeneration, resulting in increased neuronal vulnerability. Transplantation of exogenous mitochondria to the injury site mitigates cellular energy crises and counteracts neurodegeneration; however, the limited efficacy of mitochondrial transplantation alone constrains its therapeutic potential. In this study, we established a right‐sided spinal cord hemisection model at the T10 thoracic segment in rats and transplanted a methacrylate‐based gelatin (GelMA) hydrogel containing active mitochondria at the injury site to assess its therapeutic effects and underlying mechanisms. Our findings indicate that GelMA hydrogel combined with mitochondrial transplantation provides superior therapeutic benefits for SCI compared to mitochondrial transplantation alone. GelMA hydrogel enables sustained mitochondrial release at the injury site, supplying energy, upregulating NF200 expression, and promoting axonal regeneration. Additionally, it enhances M2 macrophage accumulation and improves the local inflammatory microenvironment. The structural framework of GelMA hydrogel further supports axonal regeneration. Footprint gait analysis and Basso, Beattie, and Bresnahan (BBB) motor scoring demonstrated that GelMA hydrogel combined with mitochondrial transplantation significantly improves motor function in the right hind limb of rats with SCI. Consequently, GelMA hydrogel combined with mitochondrial transplantation offers a viable and promising approach for treating spinal cord injury.

**Translational Impact Statement**

This study investigated the transplantation of mitochondria‐loaded GelMA hydrogel into rat spinal cord injury sites. The hydrogel facilitated mitochondrial release, supplying energy to cells in the injured area, which reduced cell death and promoted axonal regeneration. This novel delivery approach presents a promising therapeutic strategy for treating spinal cord injuries.

## 1 INTRODUCTION

Spinal cord injury (SCI) is a severe central nervous system disorder that typically results in varying degrees of neural function loss below the injury site.^1^ Currently, effective clinical treatments for SCI are limited. The unique characteristics of nervous tissue, along with the complex pathophysiological changes following SCI, create significant challenges in treatment. The nervous system is highly energy‐dependent, with adenosine triphosphate (ATP) production by mitochondria being essential for maintaining membrane potential, neural signaling, and axonal regeneration.^2^ To meet energy demands, neurons rely on a finely regulated mitochondrial transport system. Newly synthesized mitochondria in the soma replace damaged or aging mitochondria in distal axons through anterograde transport, while damaged mitochondria return to the soma via retrograde transport for repair or degradation.^3^ However, SCI disrupts this transport system, leading to reduced mitochondrial movement and energy deficiency, which can hinder axonal regeneration.^4^, ^5^

To address these energy deficits that impede axonal regeneration, research suggests that supplementing mitochondria in the SCI region may promote regeneration. Compared to untreated SCI models, therapeutic strategies that promote mitochondrial transfer or increase exogenous mitochondrial availability have been shown to improve motor function, reduce inflammatory responses, and apoptosis at the injury site, and facilitate axonal growth.^6^, ^7^, ^8^ However, mitochondrial transplantation alone has limitations. Transplanted mitochondria often fail to remain stable in the targeted SCI area due to cerebrospinal fluid flow, preventing maintenance of effective therapeutic concentrations. Furthermore, high concentrations of mitochondria at the injury site may reduce host cell absorption efficiency.^9^

Encapsulating mitochondria in hydrogels has emerged as a promising approach for SCI repair, as biomaterials can provide spatial support for nerve regeneration. Research has shown that isolated mitochondria encapsulated in methacrylate‐based gelatin (GelMA) hydrogel can sustain ATP production for over 24 h and exhibit slightly enhanced ATP‐generating capacity.^10^ GelMA hydrogels are synthesized from methacrylic anhydride (MA) and gelatin and polymerize through photo‐crosslinking, forming a stable structure. By adjusting light exposure, GelMA hydrogels can be tailored to meet specific transplantation and treatment needs.^11^ Studies by Hu et al. and Tao et al. have demonstrated the efficacy of combining GelMA hydrogels with reduced graphene oxide (rGO) nanosheets or custom nanoparticles to support nerve regeneration and functional recovery.^12^, ^13^ For example, GelMA‐MXene conduits containing neural stem cells improved the local inflammatory environment and promoted axonal connectivity when implanted in injured spinal cord regions.^14^ These findings indicate that GelMA hydrogels are effective in SCI treatment and can achieve favorable therapeutic outcomes. Therefore, we applied GelMA hydrogels combined with mitochondrial complexes in a rat model of SCI to evaluate their combined therapeutic effects.

## 2 MATERIALS AND METHODS

### 2.1 Experimental animals

All animal experiments were approved by the Animal Ethics Committee of Yangzhou University (202206950). Adult male Sprague–Dawley (SD) rats of SPF grade, aged 8–10 weeks and weighing 200–250 g, were obtained from Nanjing Niubang Biotechnology Co., Ltd. (Nanjing, China). Rats not meeting the experimental criteria (e.g., weakness, low motor function scores, poor nutrition) were excluded. The remaining rats were housed in an SPF‐grade animal facility with controlled temperature, humidity, and access to food and water, under an environment compliant with Institutional Animal Care and Use Committee guidelines. Rats were randomly divided into four groups: SCI + Vehicle group (blank control), SCI + GelMA group (hydrogel only), SCI + Mito group (mitochondria only), and SCI + GelMA + Mito group (hydrogel–mitochondria combination). This animal experiment adheres to the ARRIVE guidelines; animal allocation in this study was performed using a double‐blind method, where the person responsible for assigning the animals was unaware of the group allocation. Additionally, the researchers conducting post‐surgical data analysis were blinded to the group assignments of the rats.

### 2.2 Extraction of active mitochondria

Following anesthesia with 1.25% tribromoethanol (10 mL/kg, intraperitoneal), SD rats were placed prone on a heating pad. The skin over the hind limb was incised to access the gastrocnemius muscle, exposing the soleus muscle beneath. The soleus muscle was excised, placed in 4°C 1X PBS, minced, washed three times with 4°C 1X PBS, and suspended in a mitochondrial separation reagent (model: BL153A, Biosharp, Beijing, China) before grinding. The homogenate was centrifuged at 600*g* for 5 min at 4°C, and the supernatant was transferred to a clean centrifuge tube for a second centrifugation at 12,000*g* for 10 min at 4°C. The supernatant was discarded, and the resulting pellet, containing mitochondria, was suspended in pre‐chilled 1X PBS at 4°C. All steps were conducted on ice or in a 4°C environment, and solutions were pre‐chilled to 4°C. The entire extraction process was completed within 1 h. The mitochondrial suspension was finally assessed for protein concentration using a BCA assay.

### 2.3 Mitochondrial fluorescent labeling

MitoTracker Red CMXRos (model: M7512, ThermoFisher, USA) was used to fluorescently label the mitochondria. Mitochondria were stained with 200 nM MitoTracker Red at 37°C for 30 min, followed by high‐speed centrifugation to remove excess dye. The washed mitochondria were then resuspended in pre‐chilled mitochondrial storage solution. Fluorescent labeling was confirmed by observing red fluorescence under an inverted fluorescence microscope.

### 2.4 Preparation of GelMA‐Mito hydrogel composite

GelMA solution and the photoinitiator lithium phenyl‐2,4,6‐trimethylbenzoylphosphinate (LAP) were prepared in a sterile environment (Shanghai Aladdin Biochemical Technology Co., Ltd.). Following the manufacturer's protocol, 20 mg of GelMA (GM60%) was dissolved in 1 mL of PBS, along with a 0.25% LAP photoinitiator, both prepared in a 37°C water bath. Mitochondria at a concentration of 10 μg/μL were added to the GelMA solution in a 1:10 volume ratio, followed by exposure to blue light (405 nm) for 1 min to induce chemical crosslinking.

### 2.5 Scanning electron microscopy of the hydrogel

To observe the hydrogel's microscopic morphology, it was dehydrated using a drying machine and frozen overnight at −80°C. The hydrogel was then sectioned in liquid nitrogen to obtain cross‐sections, which were freeze‐dried to preserve morphology. Freeze‐dried samples were mounted on a copper stage with conductive adhesive, coated with gold, and observed under a scanning electron microscope (Zeiss) at an acceleration voltage of 5 kV.

### 2.6 Rheological tests

Rheological tests were performed on the hydrogels using an Anton Paar rheometer (Austria). Time‐sweep tests were conducted at 37°C, with a frequency of 1 Hz and a strain of 1%, recording changes in storage modulus (*G*′) and loss modulus (*G*″). Viscosity changes were also measured over a strain range of 0.1% to 1000%, with the temperature set at 37°C and the frequency fixed at 1 Hz.

### 2.7 Construction of the rat spinal cord hemisection injury model

SD rats were anesthetized with 1.25% tribromoethanol (10 mL/kg, intraperitoneal injection) and positioned prone. The skin and muscle layers were sequentially separated to expose the T10 spinous process and lamina complex, which were then removed. A midline incision of the dura mater was performed to expose the spinal cord, and 2 mm of spinal tissue to the right of the T10 segment was excised. Based on previous studies, an optimal mitochondrial injection dose of 100 μg was used.^6^, ^9^ In the SCI + Mito group, 10 μL of mitochondrial suspension (10 μg/μL) was injected into the spinal cord defect. For the SCI + GelMA group, 100 μL of hydrogel was injected; in the SCI + Mito‐GelMA group, 110 μL of a mitochondrial–hydrogel composite (100 μL hydrogel and 10 μL mitochondrial suspension at 10 μg/μL) was injected; and in the SCI group, 10 μL of 1X PBS was injected. Finally, the dura mater, muscle, and skin were sutured in layers, and the wound was disinfected. For 5 days post‐surgery, rats received daily intramuscular injections of penicillin (50.0 U/kg/day) to prevent infection, and manual bladder expression was performed twice daily, with adequate food and water provided.

### 2.8 Immunofluorescence staining

Rats were anesthetized with 1.25% tribromoethanol (10 mL/kg, intraperitoneal injection), and 0.9% saline was perfused through the heart to clear blood from the vasculature, followed by rapid perfusion with 4% paraformaldehyde. Spinal cord tissue was collected from 1 cm above and below the injury site and fixed overnight in 4% paraformaldehyde. On Days 2 and 3, the spinal cord tissue underwent gradient dehydration in 20% and 30% phosphate‐buffered sucrose solutions, with solutions changed twice daily. Successful dehydration was confirmed by the spinal cord tissue sinking to the bottom of the sucrose solution. For tissues designated for Western blot analysis, only 0.9% saline was perfused, and spinal cord tissue was collected from 1 cm above and below the injury site and stored immediately at −80°C. After dehydration, the dura mater and dorsal root nerves were removed using a dissection microscope, and sagittal sections of the spinal cord were prepared at a thickness of 15 μm using a cryostat (Leica, Germany). Tissue sections were washed with PBS (3 × 10 min), blocked for 30 min, and incubated overnight with primary antibodies at 4°C. The primary antibodies used included: anti‐myelin basic protein (1:100, A11162, ABclonal, China), anti‐neurofilament 200 (1:100, A19084, ABclonal, China), and anti‐arginase‐1 (1:100, A23648, ABclonal, China). The following day, sections were incubated at room temperature for 30 min with a secondary antibody (Goat anti‐Rabbit/Mouse polymer HRP, BR009, Signalway Antibody, USA), and the nuclei were counterstained with DAPI. Images were acquired using a confocal microscope (Zeiss, Germany); nuclear staining was performed using DAPI, excited with a 405 nm laser at a power setting of 1%–3%, with a detector gain of 500–700 and a pixel dwell time of 2–6 μs. Alexa Fluor 488 (green) and Alexa Fluor 555 (red) were excited using 488 nm and 561 nm lasers, respectively, with laser power set to 2%–6% and a pixel dwell time of 4–8 μs. Thresholds were manually set in Fiji to identify regions of interest (ROIs) and eliminate background noise. All images derived from the same experiment were processed using identical thresholding criteria to ensure consistency in analysis. The approximate threshold ranges were as follows: 30–80 for the DAPI channel, 60–120 for the FITC channel, and 80–150 for the Cy3 channel. Threshold values were determined based on visual inspection and were consistently applied across all images within the same experimental group to ensure comparability of results.

### 2.9 Western blotting

Protein concentrations were measured using a BCA assay kit. Target proteins were boiled, subjected to electrophoresis, and transferred onto a PVDF membrane. The membrane was then blocked with 5% non‐fat dry milk for 2 h. Following blocking, the membrane was incubated overnight at 4°C with the primary antibody and then with the secondary antibody for 1.5 h at room temperature. Visualization was conducted using the ChemiDoc Touch Imaging System (Bio‐Rad, USA), and gray values of the target protein were analyzed using Fiji software. The specific antibodies used included: anti‐neurofilament 200 (NF200, 1:300, A19084, ABclonal, China), anti‐arginase‐1 (Arg1, 1:2000, A23648, ABclonal, China), anti‐dynamin‐related protein 1 (Drp1, 1:1000, A17069, ABclonal, China), anti‐Inducible Nitric Oxide Synthase(iNOS, 1:500, sc‐7271, Santa), HRP‐conjugated Affinipure Goat Anti‐Rabbit IgG (H + L) (SA00001‐2, 1:5000, Proteintech), HRP‐goat anti‐mouse IgG (H + L) (BF03001, 1:10000, Biodragon), and Beta Tubulin Polyclonal Antibody (10094‐1‐AP, 1:6000, Proteintech). For detailed information, please refer to Table S2, Supporting Information.

### 2.10 Gait analysis and motor scoring scale

Basso‐Beattie‐Bresnahan (BBB) locomotor assessments were conducted on Days 7, 14, 21, and 28 post‐surgery to evaluate motor function recovery in the right hind limb of the rats. Additionally, a 1 × 1 cm grid climbing test was used to observe the movement of the ankle, knee, and hip joints. In week 4 post‐surgery, gait analysis was performed by staining the plantar surface of the hind limbs with red ink and the forelimbs with blue ink. The animals were then allowed to walk freely on white paper, measuring 20 cm wide and 50 cm long, with each rat repeating the task three times. The clearest footprint was selected to measure stride length and width of the right hind limb.

### 2.11 Data analysis

GraphPad 9 software was used for all data analyses and figure preparation. Data were presented as means ± SD. If the data followed a normal distribution, data comparisons were performed using one‐way repeated‐measures ANOVA with Tukey's multiple post hoc comparisons. If the data did not meet the assumption of normality, nonparametric tests were used, with Dunn's post hoc test applied for multiple comparisons. The differences in BBB scores among the groups were evaluated via two‐way ANOVA with Tukey's post hoc analysis. Statistical significance was considered at *p* < 0.05 (*), *p* < 0.01 (**), *p* < 0.001 (***), and *p* < 0.0001 (****).

## 3 RESULTS

### 3.1 Characterization of GelMA hydrogel

**FIGURE 1:**
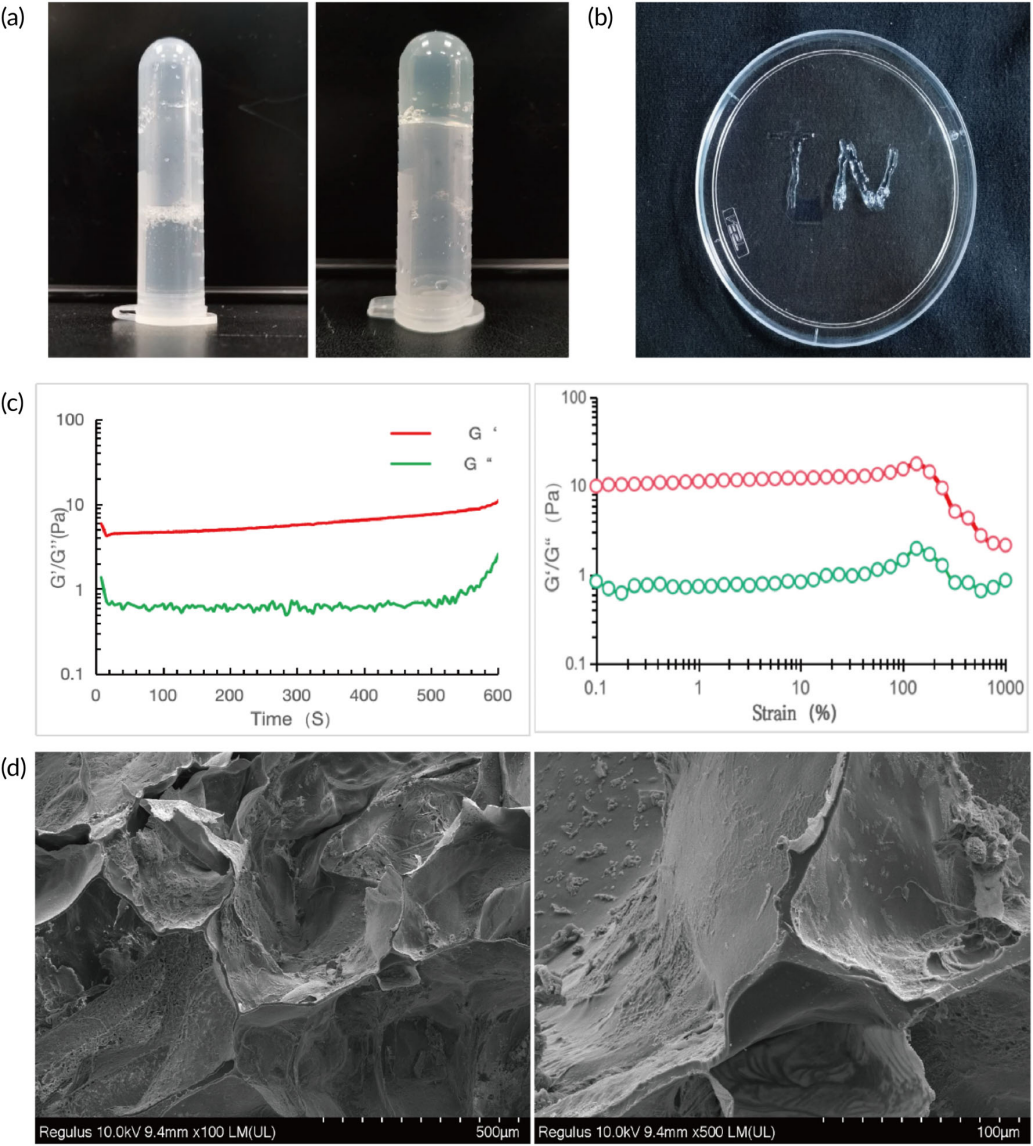
Relevant characteristics of the hydrogel appearance. (a) Left: liquid state of GelMA hydrogel; right: solid hydrogel. (b) Injectability of GelMA hydrogel. (c) Rheological testing of GelMA hydrogel: (left) under conditions of 37°C, 1 Hz frequency, and 1% strain; (right) under conditions of 37°C, 1 Hz frequency, measuring the changes in elastic modulus (*G*′) and loss modulus (*G*″) across a strain range of 0.1%–1000%. (d) SEM images of GelMA hydrogel, Scale bar: 100 and 500 μm.

A 20% (w/v) solution of GelMA hydrogel was prepared. After mixing GelMA with the LAP photoinitiator, blue light (405 nm) was applied for 1 min to induce chemical crosslinking. Successful gelation was confirmed by observing that the GelMA hydrogel remained stationary in an inverted EP tube (Figure 1a). The injectability of the GelMA hydrogel was confirmed by injecting the letters “TN” through a 26 G syringe (Figure 1b). Scanning electron microscopy revealed that the GelMA hydrogel exhibited a three‐dimensional porous network structure. This porosity meets the spatial requirements for cell growth and proliferation, aligning with essential conditions for neural cell infiltration and regeneration following spinal cord injury (Figure 1d). In addition, we also present scanning electron microscope images of GelMA hydrogel at different magnifications (Figure S1).

The elastic modulus (*G*′) and loss modulus (*G*″) of the hydrogel were quantitatively assessed using a rotational rheometer. When *G*″ > *G*′, the hydrogel is in a sol state, while *G*′ > *G*″ indicates a solid gel state. The detailed parameters for the rheological testing of GelMA hydrogel are provided in Table S1. Over time, *G*′ consistently remained greater than *G*″, with both values maintaining a stable horizontal state, demonstrating that the hydrogel does not revert to a liquid state over time (Figure 1c, left). When a strain range from 0.1% to 100% was gradually applied to the GelMA hydrogel, the values of *G*′ and *G*″ remained highly stable, confirming that the hydrogel consistently maintained its solid gel state and was unaffected by changes in internal pressure (Figure 1c, right). Rheological tests for time and pressure confirmed that the GelMA hydrogel maintained a solid gel state across defined ranges of time and pressure variations, meeting the essential requirements for its application in this in vivo experiment.

### 3.2 Sustained release of exogenous mitochondria from GelMA hydrogel

**FIGURE 2:**
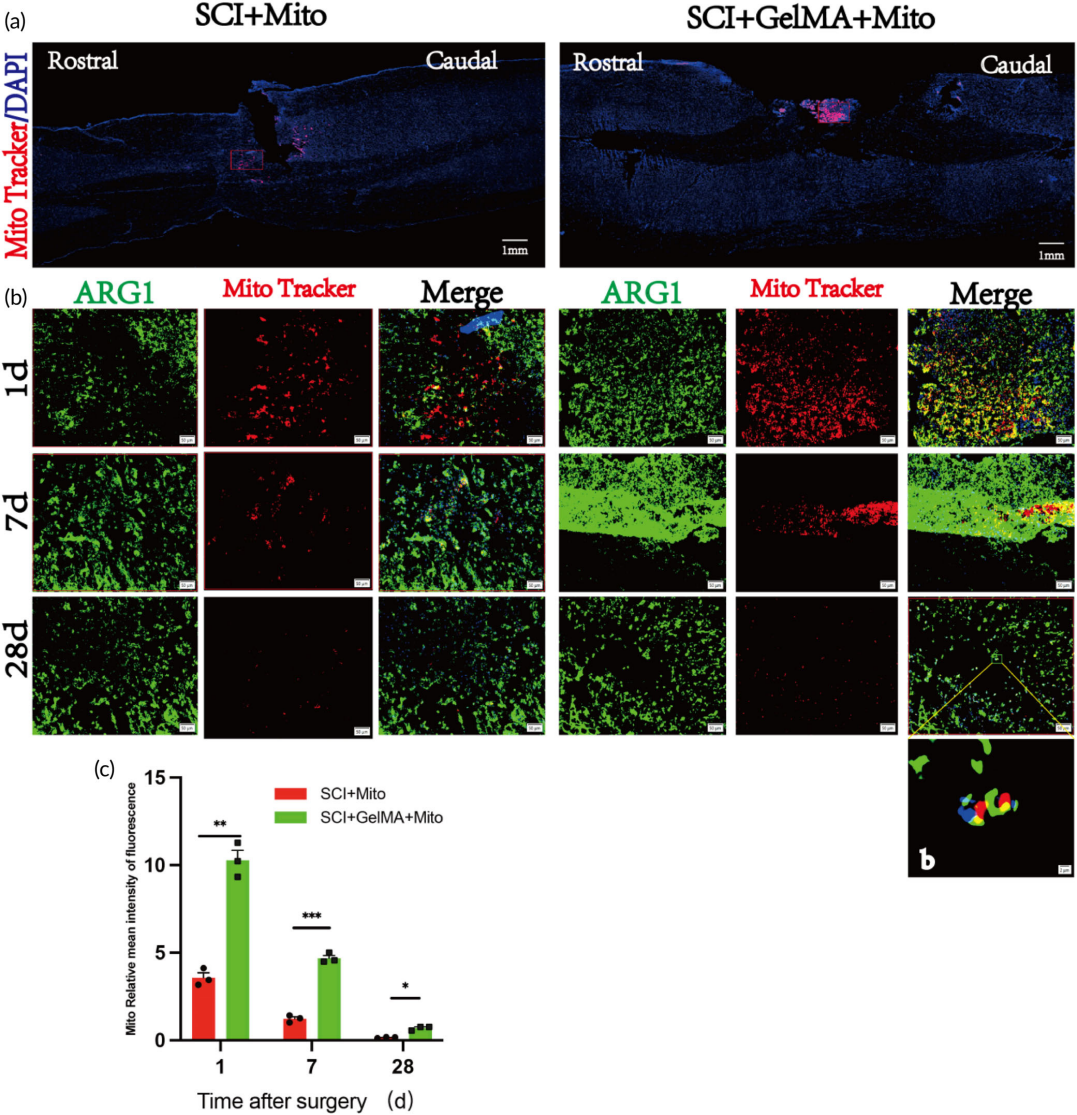
Spatial distribution and survival of exogenous mitochondria in the spinal cord. (a) Spatial distribution of exogenous mitochondria (red) at the spinal cord injury site. In the sagittal plane, exogenous mitochondria labeled with Mito Tracker Red are visible, with cell nuclei stained with DAPI (blue). Rostral = head, Caudal = tail. Scale bar: 1 mm. (b) Colocalization images of exogenous mitochondria (red) and M2 macrophages (green) at 1, 7, and 28 days post‐surgery (*n* = 3), with nuclei stained by DAPI (blue). Scale bar: 50 μm. (b) Microscopic image of exogenous mitochondria (red) being internalized by M2 macrophages (green). Scale bar: 2 μm. (c) Quantitative analysis of mitochondrial fluorescence (red) intensity at the same time points post‐surgery for the SCI + Mito and SCI + GelMA‐Mito groups, *n* = 3, *p < 0.05; **p < 0.01; ***p < 0.0001; ****p < 0.00001.

Immunofluorescence techniques were used to assess the spatial distribution and longevity of exogenous mitochondria in vivo. Spinal cord tissue was collected from euthanized rats at 1, 7, and 28 days post‐surgery to examine the distribution profile of exogenous mitochondria (red) at the injury site (Figure 2a). Overall, the SCI + Mito group displayed lower red fluorescence from mitochondria, while the SCI + GelMA‐Mito group showed a more concentrated red fluorescence at the injury site, with fluorescence intensity gradually decreasing over time (Figure 2b). On post‐operative Days 1, 7, and 28, the exogenous mitochondria in the SCI + Mito group were significantly lower than those in the SCI + GelMA‐Mito group (3.57 ± 0.488 vs. 10.284 ± 0.978 on Day 1, 1.232 ± 0.201 vs. 4.687 ± 0.28 on Day 7, and 0.154 ± 0.029 vs. 0.696 ± 0.126 on Day 28), with statistically significant differences observed (Figure 2c). GelMA hydrogel provides a supportive environment for exogenous mitochondria, thus extending their survival time. Figure 2b presents detailed images of M2 macrophages (ARG1, green) internalizing exogenous mitochondria (Mito, red), demonstrating that exogenous mitochondria can be effectively taken up by host cells.

### 3.3 Mitochondrial transplantation reduces cell death in the spinal cord injury region of rats

**FIGURE 3:**
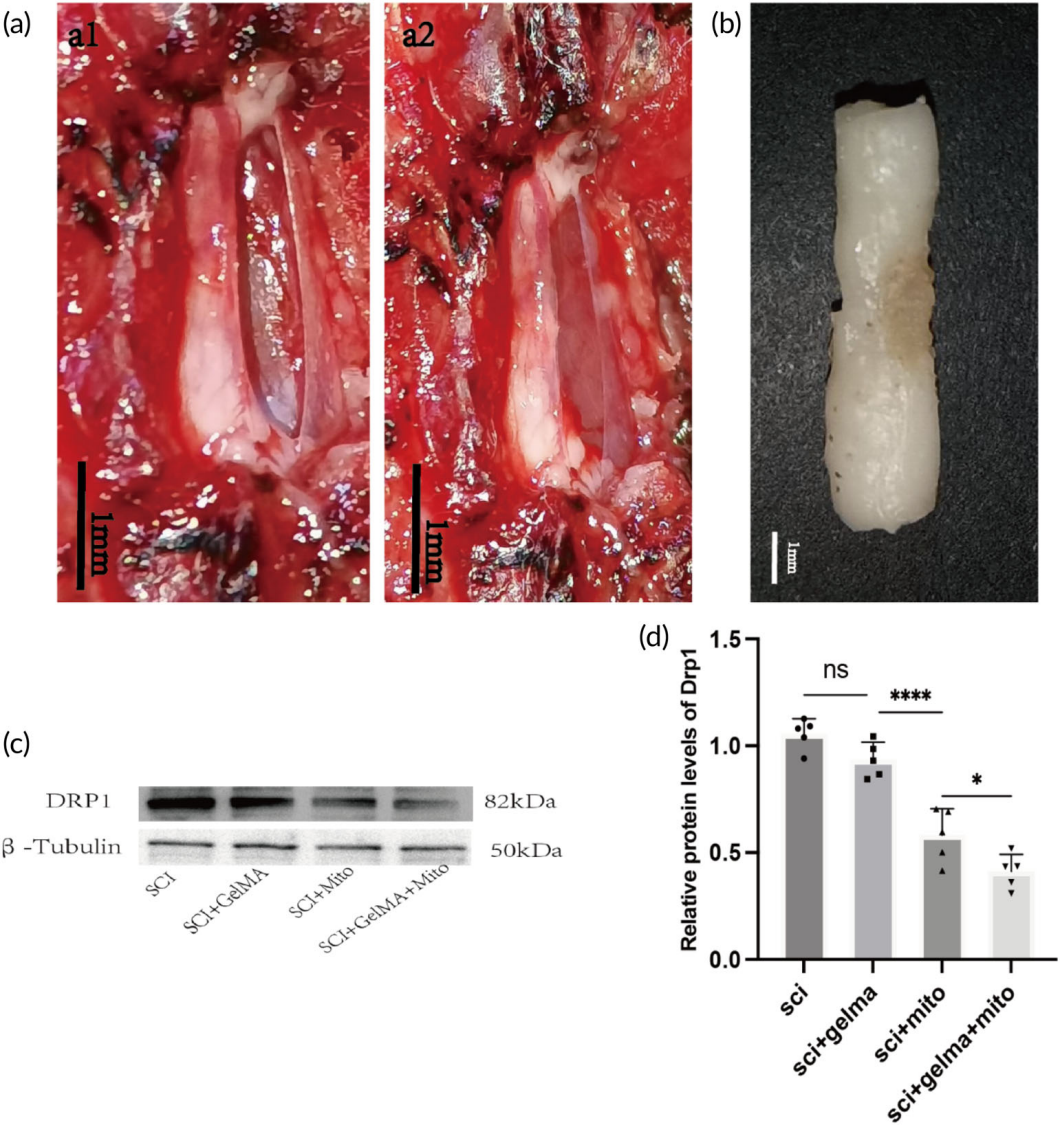
Mitochondrial transplantation improves cell apoptosis. (a) Intraoperative image of the right‐side hemisection injury model at the T10 spinal segment: (a1) No hydrogel injection. (a2) Hydrogel injection was administered. (b) Image of the dehydrated spinal cord. (c) Results of Drp1 protein expression in the center of the spinal cord injury. (d) Quantitative analysis of Drp1 protein expression in the center of the spinal cord injury, *n* = 5, *p < 0.05; **p < 0.01; ***p < 0.0001; ****p < 0.00001.

Mitochondrial fission occurs during the early stages of cell death, leading to mitochondrial fragmentation, dysfunction, and ultimately cell death.^15^ Drp1, a marker of mitochondrial fission, peaks in expression within 24 h following acute spinal cord injury.^16^ We present images from the spinal cord hemisection procedure (Figure 3a). A 2‐mm segment of the spinal cord was excised from the right side of the T10 thoracic level in rats (Figure 3a (a1)), followed by the injection of GelMA hydrogel into the resulting gap (Figure 3a (a2)). The dura mater, muscle, and skin were then sutured in sequence. Additionally, we provide images of the spinal cord following dehydration (Figure 3b), where the GelMA hydrogel is embedded within the lesion gap, serving as a bridging scaffold to support axonal regeneration. To evaluate cell death in the spinal cord injury regions, we assessed Drp1 expression levels in each group at 24 h post‐surgery using Western blot analysis (Figure 3c). Results indicated that Drp1 expression levels in the SCI + GelMA‐Mito and SCI + Mito groups were significantly lower than those in the SCI + GelMA and SCI groups, with the SCI + GelMA‐Mito group showing the lowest Drp1 expression, a difference that was statistically significant. The Drp1 protein expression level in the SCI + GelMA‐Mito group was 0.4120 ± 0.08004, which was lower than that in the SCI + GelMA group (0.9024 ± 0.09218), the SCI group (1.056 ± 0.07135), and the SCI + Mito group (0.5921 ± 0.1374) (Figure 3d).

### 3.4 GelMA hydrogel alleviates the inflammatory response at the site of spinal cord injury

After central nervous system injury, monocytes migrate to the injury site and differentiate into macrophages, peaking at Day 7 post‐injury, categorized into pro‐inflammatory M1 and anti‐inflammatory M2 subtypes.^17^ Kigerl et al. reported that M1 macrophages are neurotoxic, whereas M2 macrophages promote nerve regeneration.^18^ Additionally, Tai et al. found that inflammation in the central nervous system can impair mitochondrial function, and reducing inflammation in the spinal injury area helps improve mitochondrial function.^19^

**FIGURE 4:**
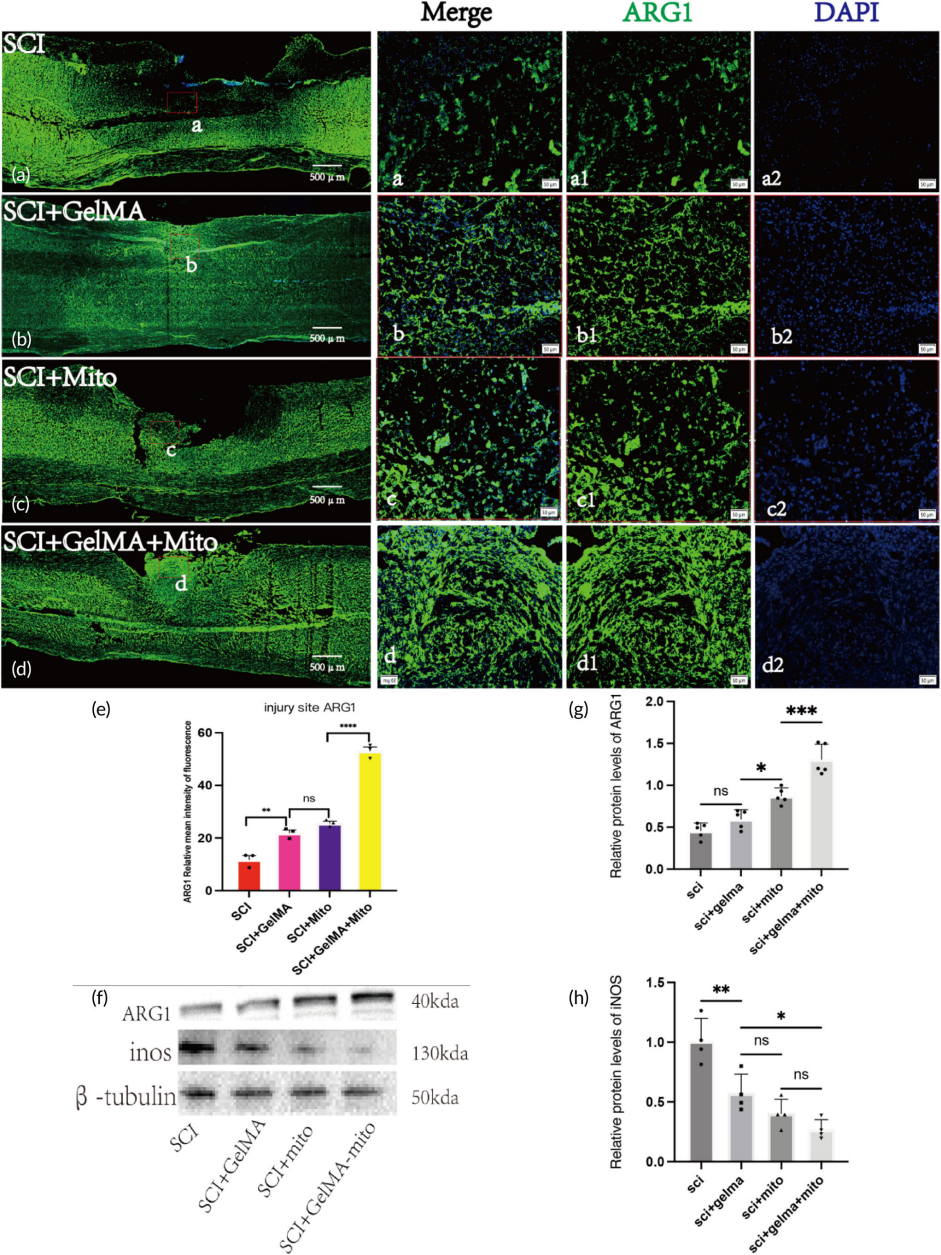
GelMA hydrogel regulates the inflammatory response 7 days post‐SCI. (a) Overall immunofluorescence staining images of ARG1 (green) in the SCI groups, Scale bar: 500 μm. (b) Overall immunofluorescence staining images of ARG1 (green) in the SCI + GelMA groups, Scale bar: 500 μm. (c) Overall immunofluorescence staining images of ARG1 (green) in the SCI + Mito groups, Scale bar: 500 μm. (d) Overall immunofluorescence staining images of ARG1 (green) in the SCI + GelMA + Mito groups, Scale bar: 500 μm. (a) Magnified views of the injury center regions from the SCI groups, Scale bar: 50 μm. (b) Magnified views of the injury center regions from the SCI + GelMA groups, Scale bar: 50 μm. (c) Magnified views of the injury center regions from the SCI + Mito groups, Scale bar: 50 μm. (d) Magnified views of the injury center regions from the SCI + GelMA + Mito groups, Scale bar: 50 μm. (a1, b1, c1, and d1) Images of ARG1 (green) positive cells in the injury center, Scale bar: 50 μm. (a2, b2, c2, and d2) DAPI (blue) staining images of cell nuclei in the injury center, Scale bar: 50 μm. (e) Quantitative analysis of relative average fluorescence intensity of ARG1, *n* = 3. (f) Protein expression results of ARG1 and INOS in the spinal injury center. (g) Quantitative analysis of ARG1 protein expression in the spinal injury center, *n* = 5. (h) Quantitative analysis of INOS protein expression in the spinal injury center, *n* = 4, *p < 0.05; **p < 0.01; ***p < 0.0001; ****p < 0.00001.

To investigate the impact of GelMA‐Mito hydrogel on inflammatory responses at the spinal injury site, we performed immunofluorescence staining for M2 anti‐inflammatory macrophages (ARG1, green) at Day 7 post‐surgery. Results showed that M2 macrophage accumulation in the GelMA‐Mito group was significantly higher than in other groups, with spinal integrity in the GelMA‐Mito and GelMA groups superior to that in the SCI + Mito and SCI groups (Figure 4a–d). Quantitative analysis of fluorescence intensity revealed that the ARG1 fluorescence intensity in the SCI + GelMA‐Mito group (53.14 ± 2.568) was significantly stronger than in the other groups, including the SCI + GelMA group (21.96 ± 1.944), the SCI group (11.84 ± 2.664), and the SCI + Mito group (25.69 ± 1.228). Additionally, the ARG1 fluorescence intensity in the groups treated with either GelMA hydrogel or mitochondria alone was also higher than that in the SCI control group, with statistically significant differences (Figure 4e). Concurrent Western blot analysis demonstrated that M2 macrophage expression levels in the SCI + GelMA‐Mito group were significantly higher than those in the other groups, with statistical significance (Figure 4f,g). The ARG1 protein expression in the SCI + GelMA‐Mito group was 1.308 ± 0.1826, which was relatively higher than that in the SCI + GelMA group (0.5973 ± 0.1111), the SCI group (0.4583 ± 0.09290), and the SCI + Mito group (0.8728 ± 0.09561). No significant difference was observed between the SCI + GelMA and SCI groups. These findings suggest that the combination of GelMA hydrogel and exogenous mitochondrial transplantation exerts a more pronounced therapeutic effect than either treatment alone. The assessment of M1 macrophage expression at the spinal cord injury site revealed that the SCI group exhibited the highest iNOS expression (1.008 ± 0.1896), which was significantly greater than that observed in the other three groups, including the SCI + GelMA‐Mito group (0.2698 ± 0.08267), SCI + Mito group (0.4028 ± 0.1205), and SCI + GelMA group (0.5732 ± 0.1592), with statistical significance. However, the administration of GelMA hydrogel or mitochondria alone demonstrated a weaker effect on inflammation modulation compared to the combined administration of GelMA hydrogel and mitochondria. No statistically significant difference was observed between the SCI + Mito group (0.4028 ± 0.1205) and the SCI + GelMA group (0.5732 ± 0.1592) (Figure 4f,h). Overall, the combined injection of GelMA hydrogel and mitochondria exhibited superior efficacy in regulating macrophage‐mediated inflammatory responses at the injury site compared to single‐agent administration.

### 3.5 GelMA‐Mito hydrogel composite promotes axonal regeneration following spinal cord injury

**FIGURE 5:**
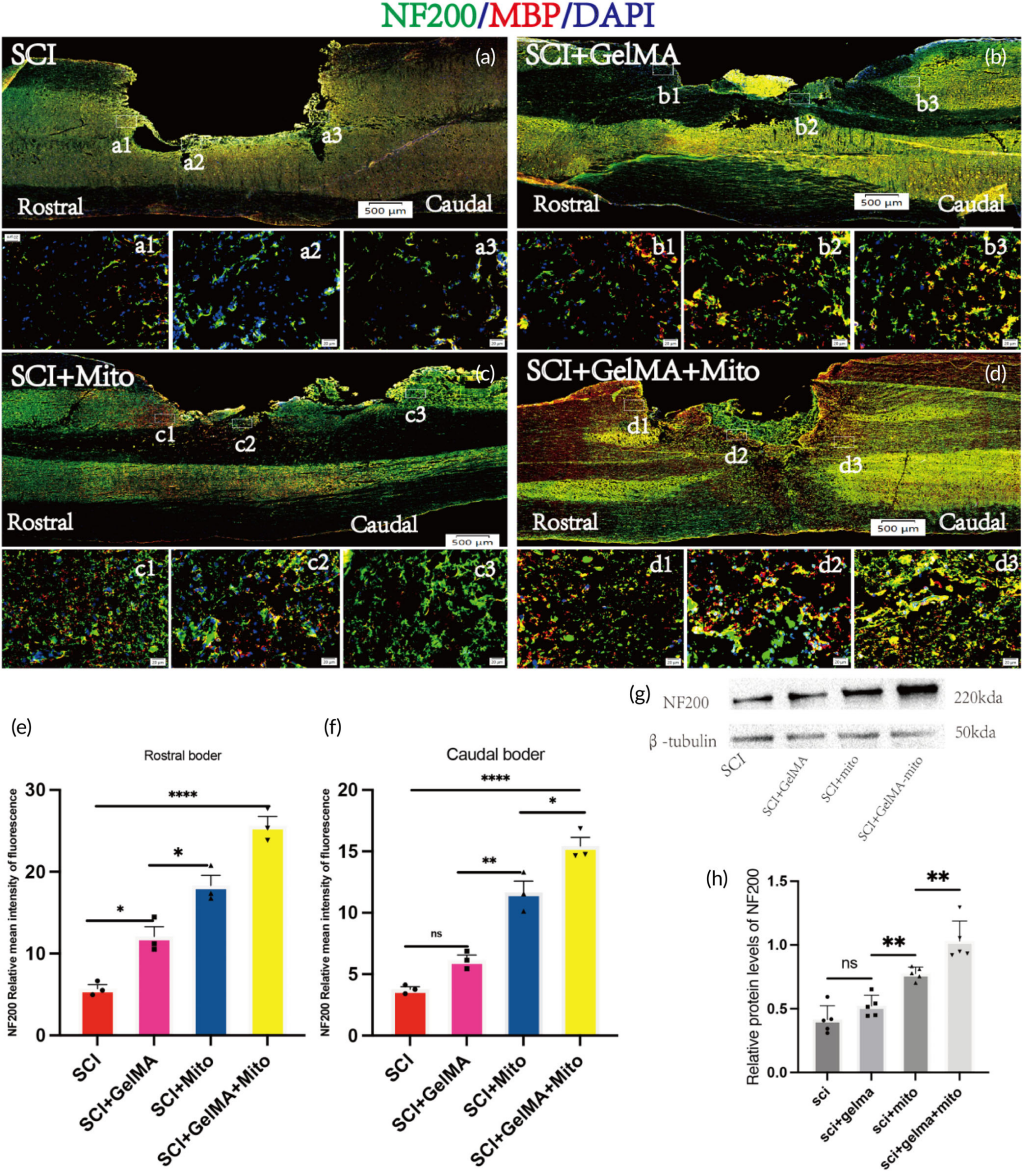
Axonal regeneration and myelination in the spinal cord at 28 days post‐SCI. (a) Overall immunofluorescence staining images of NF200 (green) and MBP (red) in the SCI groups, Scale bar: 500 μm. (b) Overall immunofluorescence staining images of NF200 (green) and MBP (red) in the SCI + GelMA groups, Scale bar: 500 μm. (c) Overall immunofluorescence staining images of NF200 (green) and MBP (red) in the SCI + Mito groups, Scale bar: 500 μm. (d) Overall immunofluorescence staining images of NF200 (green) and MBP (red) in the SCI + GelMA + Mito groups, Scale bar: 500 μm. (a1, b1, c1, and d1) are high‐magnification images of the central rostral regions of the spinal cord injury from the SCI, SCI + GelMA, SCI + Mito, and SCI + GelMA + Mito groups, respectively, Scale bar: 20 μm. (a2, b2, c2, and d2) are high‐magnification images of the injury center regions of the spinal cord injury from the SCI, SCI + GelMA, SCI + Mito, and SCI + GelMA + Mito groups, respectively, Scale bar: 20 μm. (a3, b3, c3, and d3) are high‐magnification images of the central caudal region of the spinal cord injury from the SCI, SCI + GelMA, SCI + Mito, and SCI + GelMA + Mito groups, respectively, Scale bar: 20 μm. (e, f) Quantitative analysis results of NF200 (green) fluorescence intensity in the rostral and caudal regions for the SCI, SCI + GelMA, SCI + Mito, and SCI + GelMA + Mito groups, *n* = 3. (g) Protein expression results of NF200 in the spinal injury center. (h) Quantitative analysis of NF200 protein expression in the spinal cord injury center for the SCI, SCI + GelMA, SCI + Mito, and SCI + GelMA + Mito groups, *n* = 5, *p < 0.05; **p < 0.01; ***p < 0.0001; ****p < 0.00001.

At 28 days post‐surgery, dual immunofluorescence staining of myelin basic protein (MBP) and neurofilament 200 (NF200) was performed on the spinal injury area to assess axonal regeneration in the SCI, SCI + GelMA, SCI + Mito, and SCI + GelMA‐Mito groups (Figure 5a–d). The SCI + GelMA‐Mito group exhibited stronger red and green fluorescence at the injury site, corresponding to MBP (red) and NF200 (green), compared to other groups. High‐magnification imaging of selected regions at the head, tail, and center of the injury area revealed regenerated nerve fibers closely associated with myelin. Quantitative analysis of NF200 immunofluorescence intensity at both ends of the spinal injury center indicated that, both at the rostral and caudal sides, the SCI + GelMA‐Mito group exhibited significantly higher fluorescence intensity compared to the other three groups, with statistically significant differences observed. Rostral side: SCI + GelMA‐Mito group (53.14 ± 2.568) versus SCI + Mito group (25.69 ± 1.228), SCI + GelMA group (21.96 ± 1.944), and SCI group (11.84 ± 2.664); Caudal side: SCI + GelMA‐Mito group (53.14 ± 2.568) versus SCI + Mito group (25.69 ± 1.228), SCI + GelMA group (21.96 ± 1.944), and SCI group (11.84 ± 2.664). Additionally, the NF200 fluorescence intensity at the rostral side of the injury center was higher than at the caudal side, which we speculate may be closely related to the loss of upper‐level neuronal input at the caudal side (Figure 5e,f). At the same time, the expression levels of NF200 protein indicated that the SCI + GelMA‐Mito group (1.030 ± 0.1576) exhibited a significantly higher expression than the other groups, with statistical significance, including the SCI + Mito group (0.7757 ± 0.05087), the SCI + GelMA group (0.5202 ± 0.08485), and the SCI group (0.4142 ± 0.1096). Notably, although the administration of GelMA hydrogel alone promoted axonal regeneration compared to the SCI control group, the difference did not reach statistical significance. This finding suggests that GelMA hydrogel alone may not exert an ideal therapeutic effect and instead primarily serves as a delivery vehicle for mitochondria (Figure 5g,h).

Mature neurons have a limited supply of mitochondria in the distal dendritic region, and mitochondria within the axon cannot be transported to the dendrites.^20^ Providing additional mitochondria after axonal or dendritic injury supports neuronal regeneration by supplying energy and aiding cellular repair.

### 3.6 The GelMA‐Mito hydrogel composite promotes recovery of limb motor function following spinal cord injury

**FIGURE 6:**
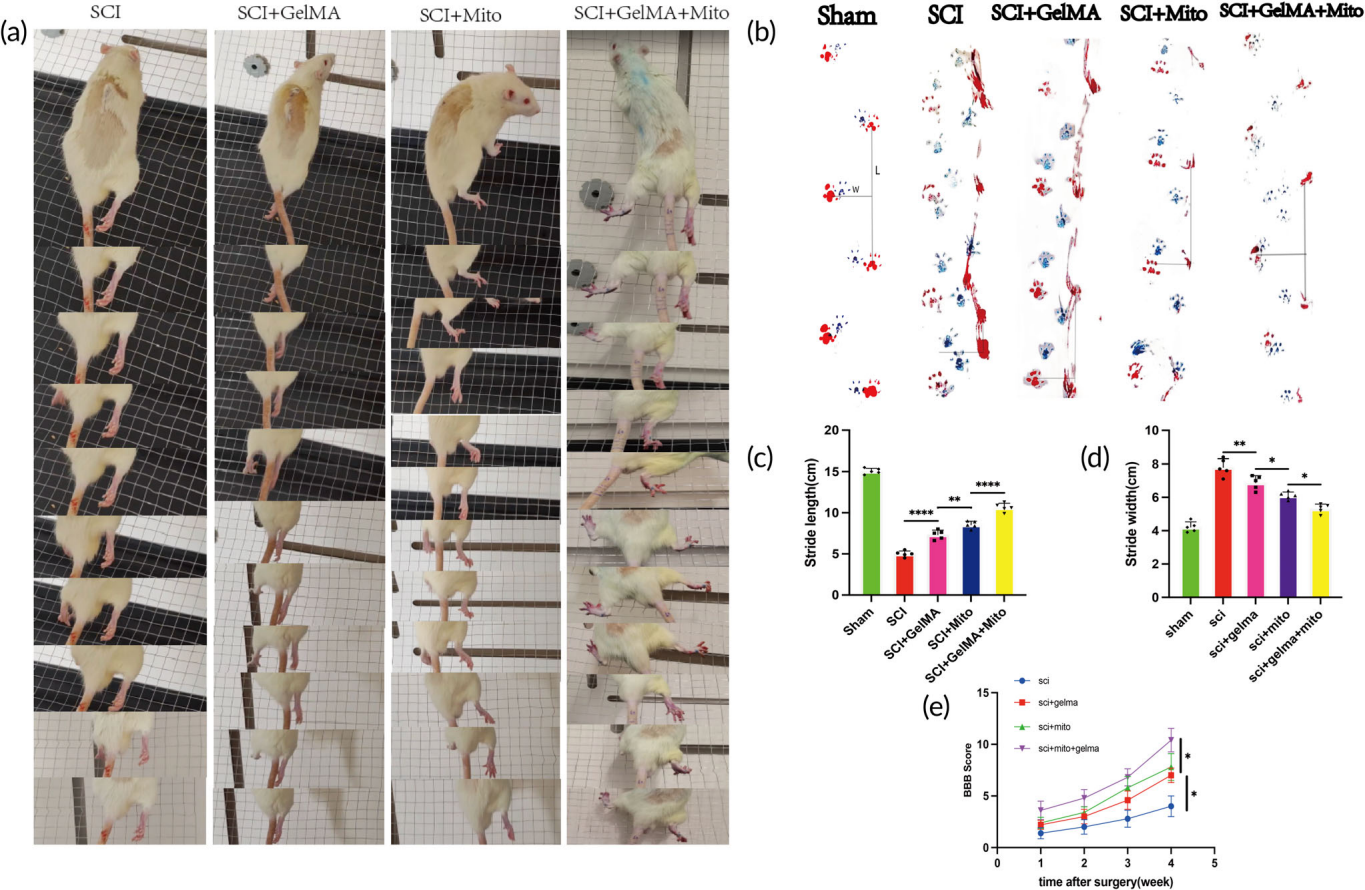
At 28 days post‐surgery, the GelMA + Mito hydrogel composite promotes the recovery of limb motor function following spinal cord injury (source: author‐generated). (a) Climbing grid images of rats in the SCI group, SCI + GelMA group, SCI + Mito group, and SCI + GelMA + Mito group post‐surgery. (b) Footprint images of normal rats, SCI group, SCI + GelMA group, SCI + Mito group, and SCI + GelMA + Mito group at 28 days post‐surgery. (c) Statistical analysis results of lateral and forward strides of the right hind limbs in rats across all groups at 28 days post‐SCI, *n* = 5. (d) Statistical analysis results of left‐right hind limb stride width in each group at 28 days post‐SCI, n = 5. (e) Statistical analysis results of BBB motor scores for rats in the SCI group, SCI + GelMA group, SCI + Mito group, and SCI + GelMA + Mito group, *n* = 5, *p < 0.05; **p < 0.01; ***p < 0.0001; ****p < 0.00001.

Immediately after surgery, the rats' right hind limbs exhibited complete paralysis, while the other limbs remained unaffected. At 28 days post‐surgery, recovery of right hind limb motor function was evaluated using a 1 × 1 cm wire mesh climbing test. In the SCI group, the right hind limb was oriented palm‐up during walking, showing poor coordination, with joints unable to flex and toes unable to grasp the wire. In contrast, rats in the SCI + GelMA + Mito group demonstrated joint flexion in the right hind limb during the test, with the palm facing downward and toes partially grasping the wire, indicating superior coordination compared to other groups (Figure 6a).

The footprint analysis results indicated that, compared to normal rats' footprints, the SCI group exhibited the widest lateral stride and the shortest forward stride of the right hind limb, whereas the SCI + GelMA + Mito group demonstrated the shortest lateral stride and the longest forward stride of the right hind limb, most closely resembling the footprint pattern of normal rats. Statistical analysis revealed the following: Stride length: SCI + GelMA‐Mito group (10.64 ± 0.5177) versus SCI + Mito group (8.540 ± 0.4393), SCI + GelMA group (7.320 ± 0.5630), SCI group (5.000 ± 0.3606), and Sham group (15.06 ± 0.3507); Stride width: SCI + GelMA‐Mito group (5.320 ± 0.2775) versus SCI + Mito group (6.100 ± 0.2236), SCI + GelMA group (6.880 ± 0.4207), SCI group (7.800 ± 0.5148), and Sham group (4.220 ± 0.3114) (Figure 6b–d). The motor function of rats was assessed using the Basso, Beattie, and Bresnahan (BBB) scoring system at 1, 2, 3, and 4 weeks post‐surgery. The SCI group did not exceed a score of 5 at Week 4, indicating limited self‐repair capacity of the spinal cord. The SCI + GelMA + Mito group exhibited the highest scores among all groups at the same time point, with a statistically significant difference at Week 4 compared to the other groups. Although the SCI + Mito group scored higher than the SCI + GelMA group, no statistically significant difference was observed (Figure 6e).

## 4 DISCUSSION

Regeneration is challenging following injury to the mature central nervous system, largely due to the inability to form new, active growth cones. The formation of growth cones requires multiple complex processes, including calcium signaling, cytoskeletal remodeling, material transport, localized mRNA translation, and the insertion of new membranes and cell surface molecules.^21^ Each of these processes demands substantial energy, making ATP critical for axonal regeneration. The presynaptic and postsynaptic terminals, active growth cones, axon branches, and nodes of Ranvier have significantly higher ATP demands than other regions of the neuron.^22^, ^23^, ^24^, ^25^ Moreover, in mature neuronal axons, most mitochondria remain stationary and cannot be transported to the distal axon and dendrites due to the anchoring mechanism mediated by SNPH.^26^ Identifying methods to disengage this anchoring mechanism could substantially aid neuronal regeneration by facilitating the transport of mitochondria to distal dendrites of mature axons, thereby providing the necessary energy for assembling new growth cones.

Following mechanical injury, excessive intracellular Ca^2+^ promotes the production of reactive oxygen species (ROS) and nitric oxide (NO). When the rate of ROS clearance is insufficient relative to its production, oxidative stress damages mitochondrial function, resulting in energy deficits.^27^, ^28^, ^29^ Impaired mitochondria cannot supply adequate energy to remove excessive intracellular Ca^2+^, leading to elevated Ca^2+^ concentrations that exacerbate mitochondrial oxidative stress.^30^, ^31^ The release of toxic ROS and apoptotic factors further worsens axonal degeneration and pathology.^32^

Studies have shown that exogenous mitochondrial transplantation regulates cell death, improves inflammatory responses, and supports neural regeneration and repair. Initially, mitochondrial transplantation was used in non‐central nervous system conditions, such as mesenchymal stem cell‐derived mitochondria transplantations for renal proximal tubular repair in diabetic nephropathy^33^ and exogenous mitochondria transplantations for drug‐induced liver injury.^34^ The significant therapeutic value demonstrated in these applications suggests the feasibility of mitochondrial transplantation for central nervous system disorders. Recently, mitochondrial transplantation has been increasingly applied to central nervous system diseases, achieving various levels of therapeutic success.^35^, ^36^, ^37^, ^38^ These studies have demonstrated promising short‐term effects in neural cell repair; however, long‐term functional recovery remains suboptimal. Thus, mitochondrial transplantation protocols require further refinement, particularly in achieving treatment stability and sustained functional improvement. Mitochondrial transplantation technology is highly adaptable and compatible, allowing it to be combined with other therapeutic strategies to enhance treatment efficacy. Advances in regenerative medicine and tissue engineering offer potential for the development of more efficient mitochondrial transplantation protocols, providing novel approaches for neurological repair. In this study, we employed a combined approach using GelMA hydrogel and mitochondrial transplantation. The localized application of the hydrogel improved the microenvironment at the injury site and provided a scaffold for cell regeneration, while the encapsulated mitochondria allowed for a more stable release, facilitating tissue repair. Our results support our hypothesis: the combination of GelMA hydrogel and mitochondrial transplantation has a more pronounced therapeutic effect on spinal cord injury repair than either treatment alone. This effect is likely due to the enhanced local microenvironment and stable mitochondrial release, which further reduce inflammation and cell death. Although the study by Westensee et al.^10^ suggests that mitochondria encapsulated in GelMA hydrogel can retain some degree of energy production capability, with improved ATP generation compared to free mitochondria, we were unable to comprehensively validate the functionality of the transplanted mitochondria in this study. Therefore, while the potential of GelMA hydrogel as a mitochondrial carrier is theoretically supported, we cannot directly conclude that the mitochondrial function was significantly improved. Future studies should include a thorough evaluation of the functionality of the transplanted mitochondria.

Exogenous mitochondrial transplantation for spinal cord injury repair has been examined using two basic delivery routes: direct injection at the lesion site and intravenous injection. Direct injection allows exogenous mitochondria to function directly on the damage site, but it necessitates an open wound, which, if not adequately managed, can exacerbate spinal cord injury. Intravenous injection is safer because it does not involve an open wound; nonetheless, exogenous mitochondria must traverse the blood‐spinal cord barrier to reach the injury site, complicating the ability to achieve appropriate therapeutic concentrations. Currently, mitochondria for spinal cord injury repair primarily originate from two sources: tissue and cells. Some studies report that cell‐derived mitochondria have 25%–50% higher viability than tissue‐derived mitochondria.^35^ However, there is no consensus on which source is optimal for spinal cord repair, and further comparative research is needed. Once exogenous mitochondria are transplanted to the spinal cord injury site, they are absorbed by various host cells. The preference of host cells for specific mitochondria and the efficiency of their absorption will be critical for optimizing mitochondrial therapies. This study did not evaluate differences in absorption rates among host cells, which is a limitation that future research should address. While some studies suggest that exogenous mitochondria are primarily absorbed by neurons,^7^ others indicate that mitochondria labeled with tGFP are mainly absorbed by macrophages.^9^, ^36^ The specific reasons for variability in mitochondrial absorption by host cells remain unclear. We hypothesize that this variability may be related to the mechanisms underlying mitochondrial uptake by different cell types. From the perspective of promoting axonal regeneration, improving the efficiency of neuronal uptake of exogenous mitochondria and specifically targeting neurons will be critical for enhancing neural recovery.

Further research is needed to develop hydrogels optimized for carrying mitochondria in transplantation. After mitochondria are transplanted with hydrogel into the spinal cord, they must be released from the hydrogel matrix to be absorbed by host cells. Excessively high concentrations of exogenous mitochondria at the injury site may reduce the efficiency of cellular absorption.^11^ Therefore, an ideal hydrogel should provide stable and sustained release of mitochondria. The release of therapeutic agents from hydrogels is governed primarily by two factors: the size of the therapeutic components relative to the pore size of the hydrogel, and the degradation rate of the hydrogel.^39^ When the components are significantly smaller than the hydrogel pores, release occurs more rapidly. Similarly, a hydrogel with a high degradation rate will release components quickly, while a slower degradation rate results in gradual release.^39^ The pore size of the hydrogel can be modified by adjusting the hydrogel concentration or other parameters, with specific techniques varying depending on the hydrogel's characteristics.^40^ The degradation rate of a hydrogel depends largely on its composition. Compared to synthetic materials, natural polymers often offer better degradation profiles and biocompatibility. An appropriate hydrogel for mitochondrial delivery should not only provide suitable release characteristics but also maintain biocompatibility, preserving mitochondrial bioactivity until their release. GelMA, derived from collagen, is a natural polymer and a common structural protein in the extracellular matrix (ECM), making it a promising candidate for hydrogel‐based mitochondrial delivery. This material regulates the degradation rate of GelMA hydrogels, facilitating the controlled release of mitochondria. Beyond degradation rate, several critical conditions must be met, including appropriate water content, tissue compatibility, multi‐dimensional structures, and large surface areas, to ensure efficient exchange between the implant and surrounding tissues.^41^ Spinal cord injuries often present irregular shapes, requiring hydrogels with injectability and tailored mechanical properties to conform to the injury gap, provide structural support, and establish a foundation for nerve regeneration and microenvironment reconstruction.^42^, ^43^, ^44^

In this study, we used only male rats as the experimental animal model. While male rats may offer certain advantages in some experiments, such as a reduced risk of urinary system complications due to their longer urethra, which simplifies postoperative management, we acknowledge that this choice introduces potential limitations, particularly in the interpretation and translation of the results. Gender differences may significantly influence biological responses and therapeutic outcomes. Several studies suggest that gender disparities can impact the inflammatory response in the central nervous system.^45^, ^46^ The sexual dimorphism of microglia, for instance, may alter the inflammatory responses in the central nervous system. Ghosh et al. observed that compared to male rats, microglia activated following spinal cord injury in female rats exhibited significantly diminished pro‐inflammatory responses and enhanced anti‐inflammatory activity, as evidenced by lower expression levels of iNOS and Toll‐Like Receptor 4(TLR4), and higher levels of ARG‐1 and Cluster of Differentiation 68(CD68) in microglia.^47^ Lively et al. found that male rats' microglia exhibited higher gene expression in response to Interferon Gamma (IFNγ) and Tumor Necrosis Factor Alpha (TNFα).^48^ Furthermore, gender differences also influence neural repair. Androgens appear to indirectly promote the survival of new neurons through androgen receptors (ARs), thereby regulating adult hippocampal neurogenesis in young male rats.^49^, ^50^ Male mice experience a shorter duration of neuropathic pain and reactive gliosis, and they also exhibit faster nerve regeneration after neural injury compared to female mice.^51^ Thus, using only male rats may lead to interpretations of treatment effects that favor a gender‐specific response pattern, potentially overlooking the different responses of female rats under the same conditions. Additionally, gender differences could also influence the translation of therapeutic strategies. For clinical applications, it is essential to consider that patients of different genders may respond differently to the same treatment. In the SCI model, where *n* = 3 may be insufficient to capture significant differences between treatment groups, the small sample size may fail to adequately control for biological variability, particularly the impact of individual differences on the results, which could lead to conservative interpretations and affect the reproducibility and generalizability of the findings. To minimize variability in baseline injury levels before treatment, standardized injury procedures are essential, as well as matching the body weights and baseline conditions of rats as much as possible between groups. Moreover, in future studies, we will implement more rigorous randomization protocols and strengthen pre‐ and post‐treatment monitoring to better control variability and ensure clear identification of differences between treatment groups. Considering factors such as gender, age, and weight that could influence experimental outcomes, we plan to include more diverse animal populations in future experiments, incorporating both male and female rats, to further explore the potential impact of these variables on treatment efficacy.

Current research suggests that single treatment strategies are insufficient for effective spinal cord injury repair. Future mitochondrial transplantation strategies using hydrogels should focus on stable, sustained release of mitochondria, enhancement of the local microenvironment at the injury site, and support of spatial structures conducive to axonal regeneration. A multidisciplinary approach will likely be essential for optimal treatment outcomes in spinal cord injuries. In summary, the combined use of GelMA hydrogels and localized mitochondrial transplantation, as presented in this study, offers a promising and feasible strategy for future research on hydrogel‐based mitochondrial transplantation for spinal cord repair.

## AUTHOR CONTRIBUTIONS

**Wenyong Gao**: Conceptualization; data curation; formal analysis; investigation; methodology; resources; writing – original draft; writing – review & editing. **Can Tang**: Conceptualization; methodology; resources. **Diancheng Hang**: Methodology; project administration; resources. **Hao Chen**: Methodology; resources. **Dongsheng Li**: Methodology; resources. **Youjie Yan**: Conceptualization; resources. **Wuchang Wang**: Conceptualization; resources. **Hao Peng**: Conceptualization; resources. **Xianmin Wang**: Data curation; resources. **Enpeng Zhang**: Data curation; resources. **Min Wei**: Methodology; project administration; resources. **Hengzhu Zhang**: Conceptualization; investigation; resources; supervision; writing – review & editing. **Xiaodong Wang**: Conceptualization; investigation; resources; funding acquisition; supervision; writing – review & editing.

## CONFLICT OF INTEREST STATEMENT

The authors declare no conflicts of interest.

## Supporting information


**Data S1.** Supporting Information.

## Data Availability

The data that support the findings of this study are available on request from the corresponding author. The data are not publicly available due to privacy or ethical restrictions.
