# Supplementary material for: Enhancement of spinal cord injury repair in rats using photo‐crosslinked GelMA hydrogel combined with mitochondrial transplantation
Source: Bioeng Transl Med. 2025 Jul 3;10(5):e70040. doi: 10.1002/btm2.70040 (PMC12478452; doi:10.1002/btm2.70040)
Supplement: Supplementary file 1 — Data S1. Supporting Information. [file BTM2-10-e70040-s001.docx]

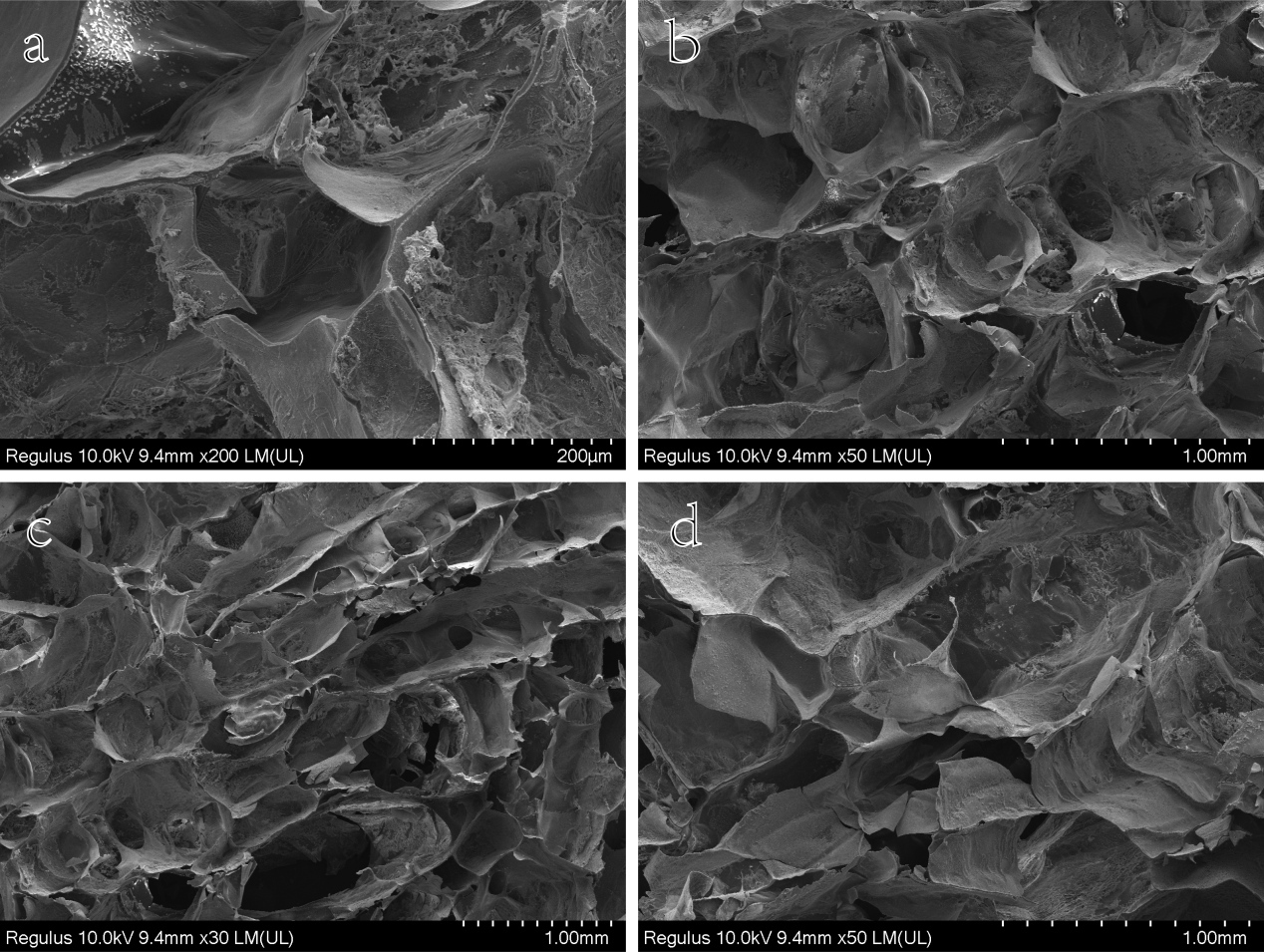


**Supplementary Figure 1**

(a, b, c and d) SEM images of GelMA hydrogel, Scale bar: 200µm and 1mm.

| **Parameter** | **Value** | |
| --- | --- | --- |
|  | Frequency-sweep | Time-sweep |
| Angular Frequency (rad/s) | 6.28 | 6.28 |
| strain | 0.1% to 1000% | 1% |
| temperature | 37°C | 37°C |
| Frequency (Hz) | 1 kHz | 1 kHz |

**Supplementary Table 1:Rheological Tests parameters of GelMA hydrogel.**

| **Antibodies used for western blot assays** | | | |
| --- | --- | --- | --- |
| Antibody name | Company | Catalog number | Concentration |
| anti-neurofilament 200 | ABclonal | A19084 | 1:300 |
| anti-arginase-1 | ABclonal | A23648 | 1:2000 |
| anti-dynamin-related protein 1 | ABclonal | A17069 | 1:1000 |
| anti-Inducible Nitric Oxide Synthase | Santa | sc-7271 | 1:500 |
| HRP-conjugated Affinipure Goat Anti-Rabbit IgG (H+L) | Proteintech | SA00001-2 | 1:5000 |
| HRP-goat anti-mouse IgG (H+L) | Biodragon | BF03001 | 1:10000 |
| Beta Tubulin Polyclonal Antibody | Proteintech | 10094-1-AP | 1:6000 |

**Supplementary Table 2. Antibodies used for western blot assays.**
